# Identification of a prognostic signature for old-age mortality by integrating genome-wide transcriptomic data with the conventional predictors: the Vitality 90+ Study

**DOI:** 10.1186/1755-8794-7-54

**Published:** 2014-09-11

**Authors:** Juulia Jylhävä, Jani Raitanen, Saara Marttila, Antti Hervonen, Marja Jylhä, Mikko Hurme

**Affiliations:** 1 Department of Microbiology and Immunology, School of Medicine, University of Tampere, FIN-33014 Tampere, Finland; 2 Gerontology Research Center, University of Tampere, Tampere, Finland; 3 School of Health Sciences, University of Tampere, Tampere, Finland; 4 UKK Institute for Health Promotion Research, Tampere, Finland; 5 Fimlab Laboratories, Tampere, Finland

**Keywords:** Mortality, Aging, Transcriptomics, Biomarkers, Cox model, Penalized regression

## Abstract

**Background:**

Prediction models for old-age mortality have generally relied upon conventional markers such as plasma-based factors and biophysiological characteristics. However, it is unknown whether the existing markers are able to provide the most relevant information in terms of old-age survival or whether predictions could be improved through the integration of whole-genome expression profiles.

**Methods:**

We assessed the predictive abilities of survival models containing only conventional markers, only gene expression data or both types of data together in a Vitality 90+ study cohort consisting of n = 151 nonagenarians. The all-cause death rate was 32.5% (49 of 151 individuals), and the median follow-up time was 2.55 years.

**Results:**

Three different feature selection models, the penalized Lasso and Ridge regressions and the *C*-index boosting algorithm, were used to test the genomic data. The Ridge regression model incorporating both the conventional markers and transcripts outperformed the other models. The multivariate Cox regression model was used to adjust for the conventional mortality prediction markers, i.e., the body mass index, frailty index and cell-free DNA level, revealing that 331 transcripts were independently associated with survival. The final mortality-predicting transcriptomic signature derived from the Ridge regression model was mapped to a network that identified nuclear factor kappa beta (NF-κB) as a central node.

**Conclusions:**

Together with the loss of physiological reserves, the transcriptomic predictors centered around NF-κB underscored the role of immunoinflammatory signaling, the control of the DNA damage response and cell cycle, and mitochondrial functions as the key determinants of old-age mortality.

## Background

Human longevity has proven to be a complex trait, and the factors enabling survival to old age are diverse. A great deal of variation also exists in the state of health in which old age is attained; some individuals age with good cognitive and physical health, whereas others suffer from multimorbidity and disabilities in daily functioning. Nevertheless, a variety of biomarkers, such as immunoinflammatory factors, endocrine mediators and indicators of functional capabilities and frailty, have been reproducibly demonstrated to be predictive of old-age survival in different populations [1-3]. In very old individuals, an elevated low-grade inflammatory state (inflammaging), which is a manifestation of immune aging, can be particularly useful for identifying those individuals at the greatest risk of mortality [1,4]. Indeed, elevated levels of conventional circulating inflammatory markers, such as interleukin 1 receptor antagonist (IL-1ra), IL-6, C-reactive protein (CRP) and tumor necrosis factor alpha (TNF-α), are among the factors that have been reported to be predictive of old-age mortality [5,6]. We have also recently identified a novel biomarker, circulating cell-free DNA, which in addition to reflecting the rate of systemic inflammation and tissue degeneration, predicts all-cause mortality in elderly individuals, independent of common risk factors, such as cardiovascular disease, dementia and diabetes [7]. Additionally, inflammaging has been regarded as a driver of the archetypal preconditions of old-age mortality, specifically neurodegeneration, muscle wasting and frailty [reviewed in [1,8]]. These observations indicate the central roles of the immune system and inflammatory functions in late-life survival.

However, only certain immunoinflammatory functions can be captured by assessing circulating inflammatory markers and determining the numbers of distinct leukocyte subsets. It is also unclear whether these parameters yield complete pictures of the biological processes that are crucial in old-age mortality. Indeed, the predictions of outcomes in patients with certain types of cancer have recently been shown to benefit from the incorporation of gene expression profiles into traditional clinical cancer risk scores [9,10]. In other potentially fatal conditions, such as stroke, the use of blood-based gene expression data in combination with other disease-associated measurements has allowed for valid classifications for the disease etiology [11]. However, the value of combining gene expression microarray data with traditional mortality predictors has not been evaluated in association with age-associated mortality. Likewise, it is largely unknown whether the changes in the gene expression patterns reported for a plethora of age-regulated transcripts [12-14] are also related to late-life mortality.

To address these issues, we sought to systematically determine the predictive performances of a wide array of conventional markers, whole-genome transcriptomic data and the combination of these data with regard to all-cause mortality. We observed that the Ridge regression model, containing the body mass index (BMI) and frailty index (conventional predictors) together with nine transcripts related to immunoinflammatory processes, cell cycle control and mitochondrial functions yielded the best-performing final signature model in terms of discriminative power and goodness-of-fit. The network analysis of the mortality-associated transcripts revealed that their actions were largely mediated through nuclear factor kappa beta (NF-κB) signaling. Thus, in addition to demonstrating the usefulness of combining transcriptomic data with conventional markers in the assessment of late-life survival, our results provide novel insights into the transcriptomic landscape preceding all-cause mortality in old age.

## Methods

### Study population

The study population consisted of n = 151 nonagenarians (n = 106 women and n = 45 men) participating in the Vitality 90+ Study, which is an ongoing study of individuals aged 90 years and older who reside in the city of Tampere, Finland. The individuals in the current study population were born in 1920 and were recruited and characterized as in the previous Vitality 90+ study cohort [5,7]. A home-visiting trained medical student performed the blood tests, physiological measurements, interviews and performance tests. Written informed consent was obtained from each participant and the study protocol followed the guidelines of the Declaration of Helsinki. The all-cause mortality data (median follow-up time of 2.55 years) including the dates of death, were collected from the Population Register Center. The mortality rate during the follow-up was 32.5%; of the151 individuals, 49 died and 102 survived the follow-up period. There were no losses to follow-up. The study protocol was approved by the Ethics Committee of the Pirkanmaa Hospital District and the Ethics Committee of the Tampere Health Center.

### RNA extraction and whole-genome transcriptomic analysis

The protocols for the leukocyte separation, RNA isolation and microarray analysis have been previously described [13,15]. Briefly, peripheral blood mononuclear cells (PBMCs) were extracted using Ficoll-Paque density gradients (Ficoll-Paque™ Premium, GE Healthcare Bio-Sciences AB, Uppsala, Sweden), after which the cells were stored at -70°C in RNAlater solution (Ambion Inc.*,* Austin, TX, USA). Following RNA extraction (miRNeasy Mini Kit, Qiagen, Hilden, Germany) and amplification (Illumina TotalPrep RNA amplification Kit, Ambion Inc.*,* Austin, TX, USA), the RNA was hybridized to a HumanHT-12 v4 Expression BeadChip (Cat no. BD-103-0204; Illumina, San Diego, CA, USA) and the chips were scanned using Beadscan (Illumina Inc., CA, USA). The qualities of the biotinylated complementary RNA products were assessed with the Agilent 2100 Bioanalyzer (Agilent Technologies Inc., Santa Clara, CA, USA). The validation of the microarray expression data through qPCR was performed as previously described [13]. The microarray data are available in the GEO database (http://www.ncbi.nlm.nih.gov/geo/) under accession number GSE40366.

The preprocessing of the microarray data was performed using the Chipster v2.8 software (http://chipster.csc.fi/) [16]. A box plot and density plots were constructed and principal component analyses were performed to assess the quality of the data. Using the lumi pipeline, the background was corrected with the bgAdjust.affy package, and the data were quantile-normalized and log2-transformed to achieve normality. Background noise and poor-quality data were filtered out based on expression levels (fluorescence intensities); the probes showing expression values of <5 or >100 in more than 5 (3.3%) samples per transcript were excluded from the analysis. Ingenuity Pathway Analysis (IPA, Ingenuity Systems, Redwood City, CA, US) was used to generate the networks and the statistically significant canonical pathways to which the identified mortality-associated transcripts were mapped. The IPA network generation algorithm creates networks by combining molecules (transcripts) based on the maximization of their specific connectivity, which is assessed as their interconnectedness relative to all molecules they are connected to in the Ingenuity Knowledge Base. The networks are ranked and scored based on the number of the Ingenuity Knowledge Base Network Eligible molecules they contain. In the network images, direct molecular relationships are displayed with continuous lines and indirect relationships with dashed lines. The significance of the association between the dataset and the canonical pathway is measured by IPA in two ways: i) based on the ratio of the number of molecules from the dataset that map to the pathway divided by the total number of molecules in the given pathway and ii) thorough the calculation of a Benjamini-Hochberg (B-H)-corrected p-value for multiple testing, which determines the probability that the association between the transcripts in the dataset and the canonical pathway is explained by chance alone. B-H-corrected p-values <0.05 (corresponding to 1.3 on a -log-scale) were considered to be statistically significant.

### Biochemical measurements and flow cytometry

The methods used to measure the plasma cf-DNA levels, unmethylated cf-DNA levels, *Alu* repeat cf-DNA plasma mitochondrial copy numbers, and CRP, IL-6, and IL-10 levels have been previously described [15]. The techniques for the determination of the plasma levels of IL-1β, IL-7, cortisol, dehydroepiandrosterone sulfate (DHEAS), indoleamine 2,3-dioxygenase (IDO) activity and anti-Epstein Barr virus (EBV) and anti-cytomegalovirus (CMV) antibody titers are described in the Additional file 1. Immunosenescence was assessed as the proportions of CD4 + CD28- cells and CD8 + CD28- cells and the ratio of CD4+ and CD8+ cells. The flow cytometric analysis used for the determination of immune cell proportions has been described in detail elsewhere [17]. Briefly, PBMCs were labeled with FITC-CD14, PerCP-Cy5.5-CD3, APC-CD28 (eBioscience, San Diego, CA, USA), PE-Cy™7-CD4 and APC-Cy™7-CD8 (BD Biosciences, Franklin Lakes, NJ, USA). The results were analyzed using the BD FACS Diva software, version 6.1.3 (BD Biosciences, Franklin Lakes, NJ, USA).

### Assessments of physiological characteristics, functional performance and frailty

The techniques used to assess the anthropometric characteristics and functional performance, i.e., the Barthel index, handgrip and Mini-Mental State Examination (MMSE), have been previously described [5,18]. The ability to perform the chair-rise test (yes/no) was assessed as the ability to stand up once from a straight-backed, regular-height chair without the use of the arms, whereas the ability to perform the chair-stand test (yes/no) was assessed as the ability to stand up and sit down five consecutive times from a straight-backed, regular-height chair. The method for determining the frailty score, which was based on criteria outlined by Fried et al. [3], has been described elsewhere [15]. The frailty index for each individual was assigned based on the frailty score as follows: 0 points = non-frail, 1–2 points = pre-frail, and 3–5 points = frail. Blood pressure was measured in a sitting position using OMRON M4 Automatic Sphygmomanometer. The mean of two consecutive measurements was considered to be the final value.

### Statistical analyses

**Figure 1:**
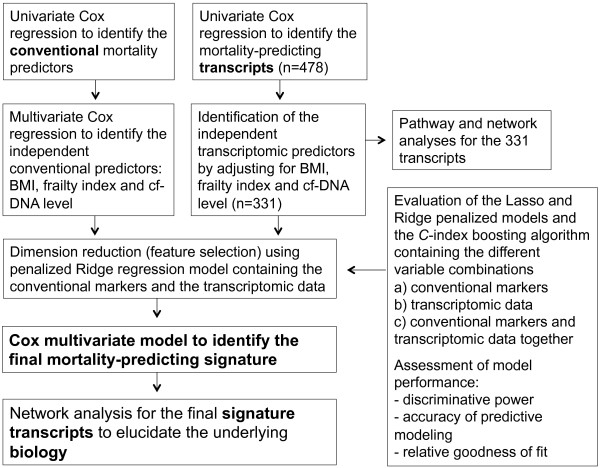
Outline of the assessment procedure for the mortality-prediction signature.

The characteristics of the study population are presented in Table 1. Each parameter presented in Table 1 was tested for its association with mortality through a univariate Cox regression analysis. A multivariate Cox regression model was fitted for all significant univariate predictors in Table 2 (left side) using stepwise selection to eliminate non-significant variables at the p = 0.05 level. The conventional variables predicting mortality in the Cox multivariate model were BMI, the frailty index and the cf-DNA level (Table 2, right side). An outline of the assessment procedure for the mortality-predicting signature is presented in Figure 1.

**Table 1:** Characteristics of the study population

	**Non-survivors**		**Survivors**	
**Variable**	**Mean/Median**	**SEM/IQR/%**	**Mean/Median**	**SEM/IQR/%**
Women (n/%)	36	73.5	70	68.6
Age (months)	1079.7	0.52	1079.9	0.32
Systolic blood pressure (mmHg)	141	3.75	150	2.92
Diastolic blood pressure (mmHg)*	70.5	14.5	74.5	19.0
Weight (kg)	63.3	1.96	70.0	1.32
BMI (kg/m^2^)	24.8	0.67	27.1	0.46
Waist circumference (cm)	89.4	1.93	94.0	1.25
Hip circumference (cm)*	98.5	11.0	102	11.5
MMSE*	24.0	7.0	26.0	4.0
Barthel index*	95.0	20.0	97.5	5.0
Handgrip (kg)*	18.0	10.5	20.0	6.5
Able to perform chair-rise test (n = yes/%)	29	63.0	82	80.4
Able to perform chair-stand test (n = yes/%)	32	72.7	87	86.1
Frailty index (n/%)				
Non-frail	3	6.1	35	34.3
Pre-frail	32	65.3	52	51.0
Frail	14	28.6	15	14.7
CRP level (ng/ml)*	2.2	7.5	2.0	3.2
IL-1β level (pg/ml)*	14.4	27.4	20.9	33.5
IL-6 level (pg/ml)*	4.9	3.1	3.8	3.9
IL-7 level (pg/ml)*	8.0	4.5	7.5	5.4
IL-10 level (pg/ml)*	1.56	1.23	1.52	1.62
cf-DNA level (μg/ml)*	0.92	0.21	0.87	0.17
Unmethylated cf-DNA level (μg/ml)*	0.73	0.20	0.67	0.16
Plasma mtDNA (copy number)*	4.27E^8^	2.68E^8^	3.64E^8^	2.32E^8^
*Alu* repeat cf-DNA (GE)*	80.2	62.7	66.5	38.3
DHEAS (μg/ml)*	0.25	0.48	0.24	0.29
Cortisol (ng/ml)*	133	55.9	125	60.9
IDO activity (Kyn/Trp)*	52.7	23.3	50.8	23.2
Anti-CMV antibody titer	19200	1145	19141	830
Anti-EBV antibody titer*	410	310	385	380
CD3+ cells (%)*^a^	60.9	21.5	57.0	13.8
CD4+ cells (%)^b^	62.3	2.38	63.6	1.42
CD8+ cells (%)^b^	31.0	2.21	29.2	1.33
CD4+/CD8+ cells (ratio)*	2.29	2.40	2.29	2.38
CD4+CD28- cells (%)*^c^	11.0	17.0	10.0	12.0
CD8+CD28- cells (%)*^d^	65.2	29.4	69.1	23.7
CD14+ cells (%)*^a^	9.5	8.6	9.5	6.4

*median value and IQR presented.

^a^percentage of live-gated cells, ^b^percentage of total T lymphocytes (CD3+ cells),

^c^percentage of CD4+ cells^, d^percentage of CD8+ cells.

*Abbreviations*: *BMI* body mass index, *CD* cluster of differentiation, *CMV* cytomegalovirus, *CRP* C-reactive protein, *cf-DNA* cell-free DNA, *DHEAS* dehydroepiandrosterone sulphate, *EBV* Epstein-Barr virus, *GE* genomic equivalent, *IDO* indoleamine 2,3-dioxygenase, *IL* interleukin, *Kyn* kynurenine, *MMSE* Mini-Mental State Examination, *mtDNA* mitochondrial DNA, *Trp* tryptophan.

**Table 2:** Mortality-predicting variables

	**Univariate**		**Multivariate**	
	**HR (95% CI)**	**p**	**HR (95% CI)**	**p**
Systolic blood pressure	0.99 (0.98-1.00)	0.039		
Diastolic blood pressure	0.97 (0.95-1.00)	0.031		
Weight	0.97 (0.94-0.99)	0.003		
BMI	0.90 (0.84-0.97)	0.004	0.91 (0.85-0.97)	0.007
Hip circumference	0.95 (0.92-0.99)	0.010		
MMSE	0.91 (0.87-0.95)	<0.001		
Barthel index	0.97 (0.96-0.99)	<0.001		
Handgrip	0.95 (0.91-0.99)	0.010		
Able to perform chair-rise test (ref. = no)	0.41 (0.23-0.73)	0.002		
Able to perform chair-stand test (ref. = no)	0.39 (0.22-0.71)	0.002		
cf-DNA level	5.17 (1.64-16.4)	0.005	3.82 (1.18-12.3)	0.025
Unmethylated cf-DNA level	5.28 (1.62-17.2)	0.006		
Frailty index (ref. = non-frail)				
Pre-frail	5.90 (1.80-19.3)	0.003	5.35 (1.63-17.6)	0.006
Frail	8.46 (2.43-29.5)	0.001	6.29 (1.77-22.4)	0.005

Abbreviations: *BMI* body mass index, cf-DNA cell-free DNA, *CI* confidence interval, *HR* hazard ratio, *MMSE* Mini-Mental State Examination.

The variables predicting mortality in the Cox univariate assessment are presented on the left side of the table and the variables remaining as independent predictors in the stepwise Cox multivariate model are presented on the right side of the table.

For high-dimensional predictors, such as whole-genome transcriptomic data, the traditional Cox regression model cannot be directly applied. As a general rule of thumb, the Cox model should be used only when there are a minimum of 10 events per predictor variable (EPV), or at least 5–9 EPV under certain circumstances [19]. Thus, we first used the Cox univariate selection method to test the mortality- associations of each of the 8,893 transcripts that passed the raw data preprocessing procedure. Statistical significance was set at p < 0.05; all transcripts passing this level were subjected to further modeling. Individual assessments of the transcripts revealed that 478 were significantly associated with survival. After individually adjusting these 478 transcripts for the conventional predictors (BMI, frailty index and cf-DNA level) in the multivariate Cox model, 331 transcripts remained significantly associated with mortality (p < 0.05). We then performed dimension reduction and feature selection using the Ridge and Lasso penalized regression models and the *C*-index boosting algorithm; all the significant 331 transcripts were included in the models without adjusting for multiple testing (please see the next paragraph for the model characteristics).

Using the 331 mortality-associated transcripts, we proceeded to test and utilize three different dimension reduction methods for feature selection. The Ridge regression model [20] shrinks the regression coefficients by imposing penalties on their squared values. Penalized maximum likelihood estimation in Cox regression with the Ridge penalty was introduced by Verweij and van Houwelingen [21], whereas Van Houwelingen et al. [22] proposed the use of the Cox model with a quadratic penalty to predict survival time based on transcriptomic data. The least absolute shrinkage and selection operator (Lasso) was introduced by Tibshirani [23]. Lasso shrinks regression coefficients toward zero by penalizing the sizes of the coefficients but uses absolute values instead of the squared values. Penalizing based on absolute values results in the number of estimated coefficients becoming exactly zero. When performing Lasso or Ridge regression, the tuning parameter (λ) must be determined to control for the amount of shrinkage. The optimal value of λ can be estimated through cross-validation; we chose the tuning parameter by maximizing the 10-fold cross-validated log partial likelihood. After defining the optimal λ, this value was used to obtain parameter estimates for the transcriptomic data-only model and the model containing the conventional predictors and the transcriptomic data (the combined model). The R package penalized was employed for the Lasso and Ridge regression with the “unpenalized” argument for the conventional variables in the combined model. As a third method, we tested the *C*-index boosting algorithm, which has been presented as an alternative means for the derivation of marker (gene) combinations via a gradient boosting framework and the direct optimization of the *C*-index [24].

We began the model selection process by evaluating the predictive performance of each model and the variable combinations through cross-validation, for which the data were split into training and test sets, and the differences in the deviance and an *R*^*2*^ measure based on the Brier score Brier score (iRBS) were calculated (described in the Additional file 1). This was followed by the assessment of the selected model for its Akaike Information Criterion (AIC) and Harrell’s *C* (also concordance index or *C*-index), which is a measure of the separation of two survival distributions [25]. The *C*-index is a performance characteristic for survival models, and it represents the fraction of all pairs of subjects whose predictions exhibit correct orders over the pairs that are able be ordered. The *C*-index estimates the probability that the order of the predictions of a pair of comparable subjects is consistent with their observed survival data.

The cut-off point for the absolute values of the coefficients was 0.0365 for the best-performing prediction model (Ridge regression with conventional predictors and transcriptomic data). All predictors showing a regression coefficient above the cut-off point were fitted to a multivariate Cox regression model using a stepwise selection method. The Cox model assumes proportional hazards, i.e., a log-linear relationship between the hazard rates and the independent parameters in the model during the follow-up period. The violation of the proportionality assumption during the follow-up period was assessed by extending the Cox model to incorporate time-dependent covariates representing the interactions between each of the independent parameters and the parametric function of the follow-up time. We also calculated the scaled Schoenfeld residuals for each independent parameter. Testing time-dependent covariates is equivalent to testing for a non-zero slope in a generalized linear regression of scaled Schoenfeld residuals as a function of time. A non-zero slope is an indication of a violation of the proportional hazard assumption. Based on the global test, no evidence of a statistically significant dependence of mortality on time was observed (p = 0.11). All Cox regression models were performed using the Stata software (version 13.0 for Windows, StataCorp LP, TX, USA).

## Results

The distributions of all examined variables (i.e., the conventional markers) are presented in Table 1. The conventional markers that were observed to predict mortality in the univariate and multivariate Cox regression models are presented in Table 2. Sex was not associated with mortality (p = 0.476) in Cox univariate regression in this cohort; thus, it was not included in the further models (please see Additional file 1 for the clarification behind this somewhat unexpected result). Likewise, age in moths was not associated with mortality (p = 0.654) in the Cox univariate regression model.

The 478 transcripts displaying expression levels associated with survival in the Cox univariate regression model are presented in Additional file 2: Table S1, and the 331 transcripts that remained as independent mortality predictors after adjustment for BMI, frailty index and cf-DNA level are presented in Additional file 3: Table S2. The top 10 canonical pathways to which these 331 transcripts were mapped are presented in Table 3; these pathways exhibited a preponderance of various immune signaling functions. The top-ranked network that was generated via IPA from these 331 transcripts (IPA score = 38) consisted of the *Cell death and Survival, Inflammatory Response* and *Cellular Function and Maintenance* functions (Additional file 4: Figure S1). The tested models, i.e., the Cox regression model containing the conventional markers alone and the three different feature selection models (the Lasso and Ridge regressions and the C-index boosting algorithm) were evaluated for their predictive accuracies (generalizabilities) using the deviance from the null model and iRBS. The evaluation criteria revealed that the model containing the conventional markers alone and the Ridge regression model containing both the conventional markers and transcriptomic data (i.e., the combined model) were superior to the other models, displaying the lowest median values for the deviance from the null model and the highest median values in the iRBS assessment (Additional file 5: Figure S2 and Additional file 6: Figure S3, respectively). In general, the other models in addition to the use of the transcriptomic data alone regardless of the model, performed poorly in the generalizability assessment (Additional file 5: Figures S2 and Additional file 6: Figure S3).

**Table 3:** The 10 most significant mortality-associated canonical pathways

**Ingenuity canonical pathway**	-log(p)*	**Ratio**	**Transcripts**
LPS-stimulated MAPK Signaling	2.97	0.11	*NFKBIA*, *MAP2K2*, *PIK3C3*, *RAC1*, *MAPK9*, *IKBKE*, *MAP2K3*, *ELK1*, *PRKCB*
CD28 Signaling in T Helper Cells	2.68	0.08	*CALM1* (includes others), *NFKBIA*, *MAP2K2*, *PIK3C3*, *HLA-DRA*, *RAC1*, *MAPK9*, *CD86*, *IKBKE*, *ARPC4*
B Cell Receptor Signaling	2.33	0.07	*CALM1* (includes others), *NFKBIA*, *MAP2K2*, *PIK3C3*, *RAC1*, *MAPK9*, *IKBKE*, *MAP2K3*, *INPP5K*, *ELK1*, *PRKCB*
CD40 Signaling	2.33	0.10	*NFKBIA*, *MAP2K2*, *LTA*, *PIK3C3*, *MAPK9*, *IKBKE*, *MAP2K3*
Pyridoxal 5′-phosphate Salvage Pathway	2.33	0.11	*MAP2K2*, *PIM1*, *GRK6*, *CDK6*, *MAPK9*, *MAP2K3*, *IRAK1*
Natural Killer Cell Signaling	2.33	0.08	*KIR2DL1*/*KIR2DL3*, *KIR3DL1*, *MAP2K2*, *PIK3C3*, *RAC1*, *INPP5K*, *KIR2DL4*, *SH2D1B*, *PRKCB*
IL-1 Signaling	2.22	0.08	*TOLLIP*, *NFKBIA*, *MAPK9*, *IKBKE*, *MAP2K3*, *GNA13*, *PRKAR1A*, *IRAK1*
Salvage Pathways of Pyrimidine Ribonucleotides	2.22	0.09	*NME4*, *MAP2K2*, *PIM1*, *GRK6*, *CDK6*, *MAPK9*, *MAP2K3*, *IRAK1*
CD27 Signaling in Lymphocytes	2.15	0.11	*SIVA1*, *NFKBIA*, *MAP2K2*, *MAPK9*, *IKBKE*, *MAP2K3*
PI3K Signaling in B Lymphocytes	2.15	0.07	*CALM1* (includes others), *NFKBIA*, *MAP2K2*, *FOXO3*, *RAC1*, *IKBKE*, *PLEKHA1*, *ELK1*, *PRKCB*

*Benjamini-Hochberg -corrected p-value.

The presented pathways are generated from the 331 transcripts that predicted mortality independent of BMI, frailty index and cf-DNA level.

**Figure 2:**
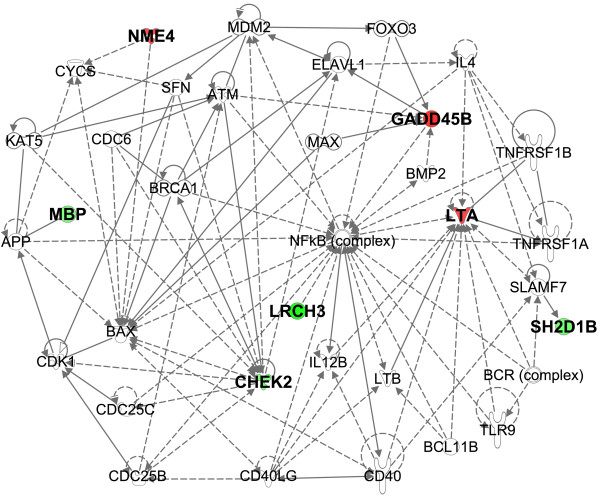
**The top-ranked IPA-generated network based on the 331 mortality-associated transcripts.** The expression levels of these transcripts predicted mortality independent of BMI, frailty index and cf-DNA level. The molecules in the network are enriched for the following functions: *Cell Death and Survival, Inflammatory Response* and *Cellular Function and Maintenance.* Green color indicates that low expression level of the transcript predicts mortality, whereas red color indicates that high expression level of the transcript predicts mortality.

We next evaluated the goodness-of-fit (AIC) and discriminative power (Harrell’s *C*) of the variable combinations, beginning with the model containing only the conventional markers and adding the Ridge regression-identified transcripts one-by-one (Additional file 7: Table S3). The rankings and regression coefficients for the combined Ridge regression model are presented in Additional file 8: Table S4. Notably, marked improvements were observed in the models’ discriminative powers (from 71.1% to 85.7%) and goodness-of-fit values (from 449.5 to 391.1) up to the model no. 15 following the addition of the transcriptomic predictors (Additional file 7: Table S3).Therefore, model no. 15 was considered to be the final mortality signature. The stepwise Cox regression analysis of the final signature demonstrated that high expression levels of *lymphotoxin alpha* (*LTA*), *NME/NM23 nucleoside diphosphate kinase 4* (*NME4*) and *growth arrest and DNA-damage-inducible beta* (*GADD45B*) and low expression levels of *myelin basic protein* (*MBP*), *SH2 domain containing 1B* (*SH2D1B*), *checkpoint kinase 2* (*CHEK2*), *leucine-rich repeats and calponin homology domain containing 3* (*LRCH3*), *transmembrane protein 70* (*TMEM70*) and *vitamin K epoxide reductase complex, subunit 1* (*VKORC1*) together with a low BMI and increased frailty were the most predictive of mortality (Table 4). The highest-ranking IPA-generated network incorporated 7/9 of the final signature transcripts and consisted of the following functions: *Cell Cycle, Cell Death and Survival,* and *Hematological System Development and Function* (Figure 2).

**Table 4:** The final mortality-predicting signature assessed using the Cox multivariate regression model

	**HR (95% CI)**	**S.E.**	***Z***	**p**
BMI	0.84 (0.77-0.91)	0.03	-4.22	<0.001
Frailty index (ref. = non-frail)				
Pre-frail	9.53 (2.70-33.6)	6.12	3.51	<0.001
Frail	17.7 (4.61-67.9)	12.14	4.19	<0.001
*TMEM70*	0.39 (0.18-0.84)	0.15	-2.40	0.017
*GADD45B*	2.60 (1.02-6.62)	1.24	2.00	0.045
*NME4*	1.77 (1.11-2.80)	0.41	2.42	0.015
*MBP*	0.58 (0.36-0.93)	0.14	-2.27	0.023
*CHEK2*	0.26 (0.12-0.55)	0.10	-3.50	<0.001
*VKORC1*	0.33 (0.18-0.59)	0.10	-3.68	<0.001
*LRCH3*	0.47 (0.25-0.87)	0.15	-2.40	0.016
*LTA*	2.09 (1.37-3.19)	0.45	3.43	0.001
*SH2D1B*	0.52 (0.36-0.76)	0.10	-3.44	0.001

*Abbreviations*: *BMI* body-mass index, *CI* confidence interval, *HR* hazard ratio, S.E., standard error.

## Discussion

The prediction of mortality in very elderly individuals has traditionally relied upon markers reflecting immunoinflammatory and endocrine functions and parameters involving physiological capabilities. In this study, we demonstrated that integrating gene expression data into a model containing these traditional predictors results in the improved prediction of old-age mortality in terms of the discriminative power and goodness-of-fit of the model. However, among the tested feature selection methods, only the Ridge regression model performed satisfactorily in the generalizability assessment; therefore, it was selected as the preferred method for survival-signature modeling. In addition to providing a means of avoiding overfitting, obtaining a parsimonious variable set through the penalized Ridge regression was necessary to assess the relative strengths of the conventional markers and the transcripts in the final model. Among the conventional markers, both the BMI and the frailty index remained in the final model, indicating that the concomitant loss of physiological reserves in multiple homeostatic systems is detrimental to survival. However, the cf-DNA level, which appears to be the best plasma-based mortality predictor according to current data as well as our earlier Vitality 90+ cohort [7], was replaced by the transcripts in our final model, suggesting that the information captured by the cf-DNA level overlaps with and is better reflected by the transcript expression levels. The finding that none of the traditional markers of inflammaging or of T cell immunosenescence were predictive of mortality was somewhat unexpected. However, the final signature transcripts *LTA* (high expression) and *SH2D1B*, *MBP* and *LRCH3* (low expression) were ascribed to immunoinflammatory processes. Specifically, LT-α (the protein product of *LTA*), which is a member of the TNF superfamily that plays pivotal roles in the function and development of the immune system, has been regarded as a central player in various inflammatory conditions [26]. The adapter molecule SH2D1B (also known as EAT-2) is known to play an indispensable role in natural killer (NK) cell activation and cytotoxicity, and it also enhances antigen-specific immune responses [27]. For MBP of immune cell origin (termed Golli-MBP), the only function demonstrated thus far is the negative regulation of T cell activation through the inhibition of Ca^2+^ influx; the ablation of Golli-MBP leads to T cell hyperproliferation in the first phase but may subsequently trigger T cell anergy [28]. The functions of LRCH3 are poorly understood, although a recent study identified it as a TNF-α, IL-1β and EBV latent membrane protein 1-dependent upstream regulator of NF-κB activity [29]. Interestingly, the IPA-generated networks from the final signature transcripts (Figure 2) and the 331 mortality-predicting transcripts (Additional file 4: Figure S1) also displayed NF-κB as a central node, underscoring the role of NF-κB-mediated immunoinflammatory regulation in late-life mortality. Finally, as 8/10 of the mortality-associated pathways (Table 3) were assigned to functions involving adaptive and innate immunity, it appears that pervasive immunoinflammatory dysregulation at the transcriptomic level precedes old-age mortality, regardless of its cause.

The final signature transcripts *GADD45B* (elevated), *CHEK2* (decreased), *TMEM70* (decreased) and *NME4* (elevated) demonstrated that the control of the DNA damage response, apoptosis and cellular maintenance, including mitochondrial functions, were likewise essential to mortality. In addition to serving as a crucial regulator in of immune cell differentiation and cytokine production, GADD45β is upregulated under conditions of growth arrest due to cellular (genotoxic) stress and DNA damage [30]. The cell cycle checkpoint regulator CHEK2 is likewise involved in the DNA damage response by preventing the entry into mitosis following DNA damage [31]. Interestingly, CHEK2 also has a crucial role in the assembly of the mitotic spindle and the maintenance of chromosomal stability [31]. Both NME4 (also known as NDPK-D) and TMEM70 are localized to the mitochondrion. NDPK-D plays a role in the supply of nucleotides and also putatively acts in apoptosis though cardiolipin transfer [32], whereas TMEM70 is required to maintain the activity of ATP synthase [33]. In addition, the finding that the IPA-generated network based on the 331 mortality-predicting transcripts (Additional file 4: Figure S1) included the *Cell Death and Survival* and *Cellular Function and Maintenance* functions, further recapitulates the relevance of these processes in old-age mortality. Interestingly, the final signature also incorporated VKORC1 (decreased), which is an enzyme that aids in the maintenance of hemostasis through the conversion of vitamin K to its active from. However, the significance of *VKORC1* expression in immune cells in relation to mortality is ambiguous. One plausible link could be the role of vitamin K as a cofactor in posttranslational protein modification, leading to the production of γ-carboxyglutamate/vitamin K-dependent (VKD) proteins. Indeed, the VKD protein GAS6 has been demonstrated to play a role in leukocyte migration and proliferation, phagocytosis and apoptosis [34]. Alternatively, the cofactor-independent immunomodulatory activities of vitamin K might account for this finding because vitamin K has been shown to downregulate the production of certain proinflammatory cytokines – an effect potentially mediated through NF-κB [35].

In addition to the present study, one previous study performed a penalized regression analysis (Lasso) to predict age-associated mortality using transcriptomic data from cultured lymphoblastoid cell lines [36]. Despite the apparent differences in the settings of these studies, the cellular functions represented by the top-ranking transcripts were similar. For example, the most significant survival-associated transcripts found in the study by Kerber et al., [36] were *CORO1A*, *IQGAP1*, *AURKB*, *TERF2IP* and *CBX5*, which play roles in processes such as T-cell mediated immunity, mitochondrial apoptosis, mitosis and chromatin maintenance. However, a between-study comparison of the survival-associated transcripts (Additional file 2: Table S1 for our data) revealed only 12 common transcripts, of which four (*CYB5B*, *IQGAP1*, *TERF21P* and *UBEV2*) exhibited *Z*-scores of the same irectionality. This discrepancy could be at least partially due to the differences in the cell types used in these investigations, which were *in vivo* blood mononuclear cells in our study but were cultured and transformed B cells in that of Kerber et al. [36].

Using another type of approach, van den Akker et al. [37] conducted a meta-analysis on established aging-associated transcripts and identified a protein-protein interaction module (Module F) consisting of 33 transcripts, whose mean expression was also associated with old-age survival in the Leiden Longevity Study [37]. In accordance with our findings, this module contained transcripts involved in mitochondrial functions (e.g., *MTERF*, *ACADM* and *TFB2M*) and the regulation of the cell cycle and mitosis (e.g., *BUB3*, *APPBP1* and *CDC23).* Four age-associated transcripts in Module F (*BUB3*, *APPBP1*/*NAE1, TFB2M* and *HNRPR*) were also observed in our dataset (Additional file 2: Table S1), all of which exhibited downregulated expression associated with an increased risk of mortality. Hence, it appears that the cellular functions that are most robustly associated with old-age survival are similar in different populations but can nevertheless be captured thorough different approaches.

Overlaying the mortality-associated pathways (Table 3) with the pathways previously reported to be regulated by age in our study population (Additional file 7: Table S3 in [13]) revealed commonalities with *CD28 Signaling in T Helper Cells*, *B Cell Receptor Signaling*, *CD40 Receptor Signaling* and *PI3K Signaling in B Lymphocytes*. However, the overlap in the transcripts themselves was negligible; only 11 (*ADM*, *FAM46C*, *GRAP*, *HIST2H2AA4*, *IER2*, *IER3*, *NACC2*, *NLRP3*, *RORA* and *SOCS3*) were both mortality-associated and age-regulated. A similar phenomenon was observed in the comparison of our mortality-associated transcripts with previously reported age-regulated transcripts [12,14]. Furthermore, some of the transcripts that were both mortality- and age-associated exhibited discrepancies in their direction of expression. For example, the elevated expression of *GRAP* was associated with an increased risk of mortality (Additional file 2: Table S1), whereas the downregulated expression of this transcript was associated with increased age [12,13]. These findings raise the question whether some of the reported age-regulated gene expression changes that have been deemed unfavorable merely because they were associated with aging are in fact intentional and advantageous in the aged body. In this scenario, deviation from this optimal gene expression pattern in the opposite direction would lead to cellular disturbance, which would be relevant to mortality. Another noteworthy observation is that one well-known life span regulator, the mTOR pathway [38], did not emerge in our pathway analysis, although a few individual components of this pathway (*PIK3C3*, *PRKCB*, *RAC1* and *RPS6KA1*) were present among the 331 mortality-associated transcripts. Thus, we hypothesize that the significance of mTOR-mediated cellular regulation subsides in the later phase of life, once an individual has already reached very old age. Overall, our results suggest that while the majority of the decisive processes are likely to be common in ageing and mortality, the actual driver genes underlying these phenomena may differ.

The major limitations of the current study are the lack of an external validation cohort, which would be ideal for assessing the universality of the transcriptomic predictors, and the small sample size. In addition, because all subjects were homogeneous in terms of age (90 years), we were unable to determine whether the identified predictors perform similarly in individuals of other (old) ages.

## Conclusions

Taken together, our systematical characterization of the determinants of old-age mortality underscores the joint impact of the decline in physiological reserves, the fidelity of immunoinflammatory processes and the control of the DNA damage response, cell cycle and mitochondrial functions. In addition, our findings corroborate the proposed roles of NF-κB in the aging process and aging-related degeneration [39], and indicate that this protein complex is central to the mechanisms underlying late-life survival. We further conclude that the incorporation of gene expression data into a model with conventional predictors could contribute to the understanding of the mechanisms underlying old-age mortality. However, because cohorts including both genome-wide transcriptomic data and mortality follow-ups are currently scarce, further studies are necessary to ascertain the universality of our results.

## Abbreviations

BMI: Body mass index; cf-DNA: cell-free DNA; iRBS: *R*^*2*^ measure based on the Brier score; IPA: Ingenuity Pathway Analysis; NF-κB: Nuclear factor kappa beta.

## Competing interests

The authors declare that they have no competing interests.

## Authors’ contributions

JJ collected the samples, designed and performed the laboratory analyses, processed the microarray data and wrote the manuscript. JR performed the statistical and bioinformatic data analyses and wrote the manuscript. SM collected the samples and performed the laboratory analyses. MJ was responsible for the Vitality 90+ study design and cohort recruitment. AH was responsible for the Vitality 90+ study design and cohort recruitment. MH supervised the work and provided the facilities and reagents. All authors read and approved the final manuscript.

## Pre-publication history

The pre-publication history for this paper can be accessed here:

http://www.biomedcentral.com/1755-8794/7/54/prepub

## Supplementary Material

Additional file 1 Additional methods. Click here for file

Additional file 2: Table S1 A table listing the 478 transcripts whose expression level were associated with survival in the Cox univariate model. Click here for file

Additional file 3: Table S2 Table listing the 331 transcripts whose expression level remained as mortality predictors in the Cox multivariate model after adjusting for BMI, frailty index and cf-DNA level. Click here for file

Additional file 4: Figure S1 Predictive accuracies based on the differences in deviance. The conventional markers-only model and the six prediction methods (50 splits into training and test datasets for each) were compared with the null model containing no covariates (the horizontal line at zero). A low value for the difference in deviance corresponds to a good predictive performance. The Lasso1, Ridge1 and BoostC1 models contain only the transcriptomic data, whereas the Lasso2, Ridge2 and BoostC2 models contain the transcriptomic data and the conventional predictors. Click here for file

Additional file 5: Figure S2 Predictive accuracies based on the iRBS. The conventional marker-only model and the six prediction methods (50 splits into training and test data sets for each) were compared to the null model with no covariate (the horizontal line at zero). A large value for the difference in iRBS corresponds to a good prediction performance. The Lasso1, Ridge1 and BoostC1 models contain only the transcriptomic data, whereas the Lasso2, Ridge2 and BoostC2 models contain the transcriptomic data and the conventional predictors. Click here for file

Additional file 6: Figure S3 The IPA-generated network based on the final signature transcripts. This network demonstrates that NF-κB is a prominent mediator of the molecular interconnections. The molecules incorporated into the final signature are shown in enlarged bold font, and the connective molecules are shown in regular font. Green color indicates that low expression level of the transcript predicts mortality, whereas red indicates that high expression level of the transcript predicts mortality. Click here for file

Additional file 7: Table S3 Displaying the stepwise assessment of the variable combinations for the final Cox regression model. Click here for file

Additional file 8: Table S4 Displaying the results of the Ridge regression model performed with the conventional predictors and transcriptomic data (the combined model). Click here for file

## References

[B1] Baylis D Bartlett DB Patel HP Roberts HC Understanding how we age: insights into inflammaging Longev Healthspan 2013 2 8 10.1186/2046-2395-2-8 24472098 PMC3922951

[B2] Shaw AC Goldstein DR Montgomery RR Age-dependent dysregulation of innate immunity Nat Rev Immunol 2013 13 875 887 10.1038/nri3547 24157572 PMC4096436

[B3] Fried LP Tangen CM Walston J Newman AB Hirsch C Gottdiener J Seeman T Tracy R Kop WJ Burke G McBurnie MA Cardiovascular Health Study Collaborative Research Group Frailty in older adults: evidence for a phenotype J Gerontol A Biol Sci Med Sci 2001 56 M146 M156 10.1093/gerona/56.3.M146 11253156

[B4] Franceschi C Capri M Monti D Giunta S Olivieri F Sevini F Panourgia MP Invidia L Celani L Scurti M Cevenini E Castellani GC Salvioli S Inflammaging and anti-inflammaging: a systemic perspective on aging and longevity emerged from studies in humans Mech Ageing Dev 2007 128 92 105 10.1016/j.mad.2006.11.016 17116321

[B5] Jylha M Paavilainen P Lehtimaki T Goebeler S Karhunen PJ Hervonen A Hurme M Interleukin-1 receptor antagonist, interleukin-6, and C-reactive protein as predictors of mortality in nonagenarians: the vitality 90+ study J Gerontol A Biol Sci Med Sci 2007 62 1016 1021 10.1093/gerona/62.9.1016 17895441

[B6] Giovannini S Onder G Liperoti R Russo A Carter C Capoluongo E Pahor M Bernabei R Landi F Interleukin-6, C-reactive protein, and tumor necrosis factor-alpha as predictors of mortality in frail, community-living elderly individuals J Am Geriatr Soc 2011 59 1679 1685 10.1111/j.1532-5415.2011.03570.x 21883115 PMC4321727

[B7] Jylhava J Jylha M Lehtimaki T Hervonen A Hurme M Circulating cell-free DNA is associated with mortality and inflammatory markers in nonagenarians: the Vitality 90+ Study Exp Gerontol 2012 47 372 378 10.1016/j.exger.2012.02.011 22406558

[B8] Hubbard RE Woodhouse KW Frailty, inflammation and the elderly Biogerontology 2010 11 635 641 10.1007/s10522-010-9292-5 20571864

[B9] Bovelstad HM Nygard S Borgan O Survival prediction from clinico-genomic models–a comparative study BMC Bioinformatics 2009 10 413 10.1186/1471-2105-10-413 20003386 PMC2811121

[B10] van Vliet MH Horlings HM van de Vijver MJ Reinders MJ Wessels LF Integration of clinical and gene expression data has a synergetic effect on predicting breast cancer outcome PLoS One 2012 7 e40358 10.1371/journal.pone.0040358 22808140 PMC3394805

[B11] Jickling GC Stamova B Ander BP Zhan X Liu D Sison SM Verro P Sharp FR Prediction of cardioembolic, arterial, and lacunar causes of cryptogenic stroke by gene expression and infarct location Stroke 2012 43 2036 2041 10.1161/STROKEAHA.111.648725 22627989 PMC3422649

[B12] Harries LW Hernandez D Henley W Wood AR Holly AC Bradley-Smith RM Yaghootkar H Dutta A Murray A Frayling TM Guralnik JM Bandinelli S Singleton A Ferrucci L Melzer D Human aging is characterized by focused changes in gene expression and deregulation of alternative splicing Aging Cell 2011 10 868 878 10.1111/j.1474-9726.2011.00726.x 21668623 PMC3173580

[B13] Marttila S Jylhava J Nevalainen T Nykter M Jylha M Hervonen A Tserel L Peterson P Hurme M Transcriptional analysis reveals gender-specific changes in the aging of the human immune system PLoS One 2013 8 e66229 10.1371/journal.pone.0066229 23776639 PMC3679079

[B14] Passtoors WM Boer JM Goeman JJ Akker EB Deelen J Zwaan BJ Scarborough A Breggen R Vossen RH Houwing-Duistermaat JJ Ommen GJ Westendorp RG van Heemst D de Craen AJ White AJ Gunn DA Beekman M Slagboom PE Transcriptional profiling of human familial longevity indicates a role for ASF1A and IL7R PLoS One 2012 7 e27759 10.1371/journal.pone.0027759 22247756 PMC3256132

[B15] Jylhava J Nevalainen T Marttila S Jylha M Hervonen A Hurme M Characterization of the role of distinct plasma cell-free DNA species in age-associated inflammation and frailty Aging Cell 2013 12 388 397 10.1111/acel.12058 23438186

[B16] Kallio MA Tuimala JT Hupponen T Klemela P Gentile M Scheinin I Koski M Kaki J Korpelainen EI Chipster: user-friendly analysis software for microarray and other high-throughput data BMC Genomics 2011 12 507 10.1186/1471-2164-12-507 21999641 PMC3215701

[B17] Marttila S Jylhava J Pesu M Hamalainen S Jylha M Hervonen A Hurme M IL-7 concentration is increased in nonagenarians but is not associated with markers of T cell immunosenescence Exp Gerontol 2011 46 1000 1002 10.1016/j.exger.2011.09.004 21964236

[B18] Tiainen K Hurme M Hervonen A Luukkaala T Jylha M Inflammatory markers and physical performance among nonagenarians J Gerontol A Biol Sci Med Sci 2010 65 658 663 20421241 10.1093/gerona/glq056

[B19] Vittinghoff E McCulloch CE Relaxing the rule of ten events per variable in logistic and Cox regression Am J Epidemiol 2007 165 710 718 10.1093/aje/kwk052 17182981

[B20] Hoerl AE Kennard RW Ridge regression: Biased estimation for nonorthogonal problems Technometrics 1970 12 55 67 10.1080/00401706.1970.10488634

[B21] Verweij PJ Van Houwelingen HC Penalized likelihood in Cox regression Stat Med 1994 13 2427 2436 10.1002/sim.4780132307 7701144

[B22] van Houwelingen HC Bruinsma T Hart AA Van’t Veer LJ Wessels LF Cross-validated Cox regression on microarray gene expression data Stat Med 2006 25 3201 3216 10.1002/sim.2353 16143967

[B23] Tibshirani R Regression shrinkage and selection via the lasso J R Stat Soc Ser B 1996 58 267 288

[B24] Mayr A Schmid M Boosting the concordance index for survival data–a unified framework to derive and evaluate biomarker combinations PLoS One 2014 9 e84483 10.1371/journal.pone.0084483 24400093 PMC3882229

[B25] Harrell FE Jr Califf RM Pryor DB Lee KL Rosati RA Evaluating the yield of medical tests JAMA 1982 247 2543 2546 10.1001/jama.1982.03320430047030 7069920

[B26] Remouchamps C Boutaffala L Ganeff C Dejardin E Biology and signal transduction pathways of the Lymphotoxin-alphabeta/LTbetaR system Cytokine Growth Factor Rev 2011 22 301 310 10.1016/j.cytogfr.2011.11.007 22152226

[B27] Aldhamen YA Appledorn DM Seregin SS Liu CJ Schuldt NJ Godbehere S Amalfitano A Expression of the SLAM family of receptors adapter EAT-2 as a novel strategy for enhancing beneficial immune responses to vaccine antigens J Immunol 2011 186 722 732 10.4049/jimmunol.1002105 21149608 PMC11119279

[B28] Feng JM Hu YK Xie LH Colwell CS Shao XM Sun XP Chen B Tang H Campagnoni AT Golli protein negatively regulates store depletion-induced calcium influx in T cells Immunity 2006 24 717 727 10.1016/j.immuni.2006.04.007 16782028

[B29] Gewurz BE Towfic F Mar JC Shinners NP Takasaki K Zhao B Cahir-McFarland ED Quackenbush J Xavier RJ Kieff E Genome-wide siRNA screen for mediators of NF-kappaB activation Proc Natl Acad Sci U S A 2012 109 2467 2472 10.1073/pnas.1120542109 22308454 PMC3289371

[B30] Vairapandi M Balliet AG Hoffman B Liebermann DA GADD45b and GADD45g are cdc2/cyclinB1 kinase inhibitors with a role in S and G2/M cell cycle checkpoints induced by genotoxic stress J Cell Physiol 2002 192 327 338 10.1002/jcp.10140 12124778

[B31] Stolz A Ertych N Bastians H Tumor suppressor CHK2: regulator of DNA damage response and mediator of chromosomal stability Clin Cancer Res 2011 17 401 405 10.1158/1078-0432.CCR-10-1215 21088254

[B32] Schlattner U Tokarska-Schlattner M Ramirez S Bruckner A Kay L Polge C Epand RF Lee RM Lacombe ML Epand RM Mitochondrial kinases and their molecular interaction with cardiolipin Biochim Biophys Acta 2009 1788 2032 2047 10.1016/j.bbamem.2009.04.018 19409873

[B33] Jonckheere AI Smeitink JA Rodenburg RJ Mitochondrial ATP synthase: architecture, function and pathology J Inherit Metab Dis 2012 35 211 225 10.1007/s10545-011-9382-9 21874297 PMC3278611

[B34] Laurance S Lemarie CA Blostein MD Growth arrest-specific gene 6 (gas6) and vascular hemostasis Adv Nutr 2012 3 196 203 10.3945/an.111.001826 22516727 PMC3648720

[B35] Booth SL Roles for vitamin K beyond coagulation Annu Rev Nutr 2009 29 89 110 19400704 10.1146/annurev-nutr-080508-141217

[B36] Kerber RA O’Brien E Cawthon RM Gene expression profiles associated with aging and mortality in humans Aging Cell 2009 8 239 250 10.1111/j.1474-9726.2009.00467.x 19245677 PMC2759984

[B37] van den Akker EB Passtoors WM Jansen R van Zwet EW Goeman JJ Hulsman M Emilsson V Perola M Willemsen G Penninx BW Heijmans BT Maier AB Boomsma DI Kok JN Slagboom PE Reinders MJ Beekman M Meta-analysis on blood transcriptomic studies identifies consistently coexpressed protein-protein interaction modules as robust markers of human aging Aging Cell 2014 13 216 225 10.1111/acel.12160 24119000 PMC4331790

[B38] Johnson SC Rabinovitch PS Kaeberlein M mTOR is a key modulator of ageing and age-related disease Nature 2013 493 338 345 10.1038/nature11861 23325216 PMC3687363

[B39] Salminen A Kaarniranta K NF-kappaB signaling in the aging process J Clin Immunol 2009 29 397 405 10.1007/s10875-009-9296-6 19408108

