# Supplementary material for: Identification of a prognostic signature for old-age mortality by integrating genome-wide transcriptomic data with the conventional predictors: the Vitality 90+ Study
Source: BMC Med Genomics. 2014 Sep 11;7:54. doi: 10.1186/1755-8794-7-54 (PMC4167306; doi:10.1186/1755-8794-7-54)
Supplement: Additional file 1 — Additional methods. [file 1755-8794-7-54-S1.docx]

**Additional file 1**

**Additional methods**

*Biochemical measurements*

Plasma IL-1β levels were determined using the Pelikine Human IL-1β enzyme-linked immunosorbent assay (ELISA) kit (Sanquin Reagents, Amsterdam, The Netherlands). Plasma IL-7 concentrations were measured using the Quantikine high sensitivity human IL-7 ELISA kit (R&D Systems, Minneapolis, MN, USA). The plasma levels of DHEAS and cortisol were assessed using commercial ELISA kits (DHEA-S ELISA and Cortisol ELISA, IBL international GmbH, Hamburg, Germany)

The anti-CMV antibody and anti-EBV antibody titers were determined using commercial ELISA kits (Enzygnost^®^ Anti-CMV/IgG, and Enzygnost^®^ Anti-EBV/IgG kits, respectively, Siemens Healthcare Diagnostics Products GmbH, Marburg, Germany). The IDO activity level was determined as the ratio of the plasma levels of trp (mmol/L) and kyn (μmol/L), which were measured by high-performance liquid chromatography as previously described (Marttila *et al.* 2010).

*Assessment of the accuracy of predictive modeling*

We used two statistical approaches (the difference in deviance and iRBS) to quantify how close the predictions were to the actual outcome. To evaluate the performances of the prediction methods, we divided the data-set randomly into two sets; the training set (101 individuals) was used for estimations and the test set (50 individuals) was used for testing the prediction capability of the estimated model. The random splitting of the data into the training and test sets was performed 50 times, and the two criteria were determined for each of the splits (yielding 50 different values for both criteria).

The difference in deviance between the fitted model and the null model containing no covariates is given by

$\hat{\delta}= -2 \{l^{\left( test \right)}\left( {\hat{\boldsymbol{\beta}}}_{train} \right)- l^{\left( test \right)}\left( \boldsymbol{0} \right)\}$,

where $l^{\left( test \right)}\left( {\hat{\boldsymbol{\beta}}}_{train} \right)$ and $l^{\left( test \right)}\left( \boldsymbol{0} \right)$ are the Cox log partial likelihoods for the test data evaluated at ${\hat{\boldsymbol{\beta}}}_{train}$ and $\boldsymbol{0}$, respectively. A small value of $\hat{\delta}$ indicates good performance.

The Brier score is a quadratic scoring rule, in which the squared differences between actual binary outcomes Y and predictions p are calculated. The calculation of the Brier score for survival outcomes is possible using a weight function, which considers the conditional probability of being uncensored over time. This enables the calculation of the Brier score at fixed time points and the creation of a time-dependent curve. A time-integrated version of the Brier score is given by

$$\mathrm{iRBS}=\frac{1}{s_{2}-s_{1}}\int_{s_{1}}^{s_{2}} R_{bri}^{2}\left( t \right)dt,$$

where *s_1_* and *s_2_* represent the lower and upper bounds of a time interval of interest. In practice, this value is obtained by computing

$$R_{bri}^{2}\left( t \right)=1-{BS}^{c}\left( t \right)/{BS}_{0}^{c}(t)$$

over a fine grid of the time-points *t* ϵ [s_1_,s_2_] and then obtaining the average of these values (Bøvelstad & Borgan 2011). A large iRBS value corresponds to a good prediction performance. The R packages penalized, survival and SurvAUC were used for calculating the differences in the deviance and Brier scores.

*Examination of the lack of association between sex and mortality*

The most likely explanation for the lack of association between sex and mortality is that the men in this cohort were on average in better condition and possessed better functional capabilities compared with the women, and this phenomenon compensated for the generally higher risk of mortality observed in elderly men. Specifically, there was a smaller proportion of frail individuals among the men compared with the women (31.58% in men vs. 47.92% in women) and the opposite was observed in the non-frail category; there was a greater proportion of non-frail individuals among the men than the women (68.42% in men vs. 52.08% in women, Pearson chi^2^ p=0.244). The men this cohort also had higher median Barthel index scores (93.56 in men vs. 90.52 in women, Mann-Whitney p=0.076) and MMSE test scores (25.20 in men vs. 23.66 in women, Mann-Whitney p=0.053). Although these differences were of borderline statistical significance, the results indicate that the men and women in this cohort differed with respect to their functional capabilities at the time that the entered into the study. Because being non-frail and having higher MMSE and Barthel scores were protective against mortality in our study (Table 2), we suggest that the above-described sex-specific differences largely explain the fact that male sex was not observed to be a statistical risk factor of mortality.

*Examination of the linear relationship between BMI and mortality*

Due to the potential existence of a U-shaped relationship between mortality and BMI, we examined whether this relationship of BMI with mortality would deviate from linearity in our cohort. We assessed this by adding both a linear BMI and a 3-class BMI (the variable converted into tertiles) simultaneously to the Cox regression model. If the relationship was non-linear (e.g. U-shaped), then both the linear and 3-class BMI would remain in the model. However, only the linear variable remained as a predictor (linear BMI: HR= 0.806, SE=0.064, z =-2.70, p=0.007, CI=0.689-0.943; 3-class BMI middle tertile, ref. lowest tertile: HR= 2.168, SE=1.134, z =1.48, p=0.139, CI=0.777-6.046; 3-class BMI highest tertile, ref. lowest tertile: HR= 4.515, SE=0.452, z =1.51, p=0.132, CI=0.635-32.095). These results indicate that the relationship between mortality and BMI was linear in our cohort.

Similar tests were performed for waist circumference, hip circumference and systolic and diastolic blood pressures, none of which displayed non-linear relationship with mortality (data not shown).

**References**

1. Marttila S, Jylhävä J, Eklund C, Hervonen A, Jylhä M, Hurme M: **Aging-associated increase in indoleamine 2,3-dioxygenase (IDO) activity appears to be unrelated to the transcription of the IDO1 or IDO2 genes in peripheral blood mononuclear cells.** *Immun Ageing 2011*, **8:**9.
2. Bøvelstad HM, Borgan O: **Assessment of evaluation criteria for survival prediction from genomic data.** *Biom J 2011*, **53:**202-16.
