# Supplementary figures and images for: Identification of a prognostic signature for old-age mortality by integrating genome-wide transcriptomic data with the conventional predictors: the Vitality 90+ Study
Source: BMC Med Genomics. 2014 Sep 11;7:54. doi: 10.1186/1755-8794-7-54 (PMC4167306; doi:10.1186/1755-8794-7-54)

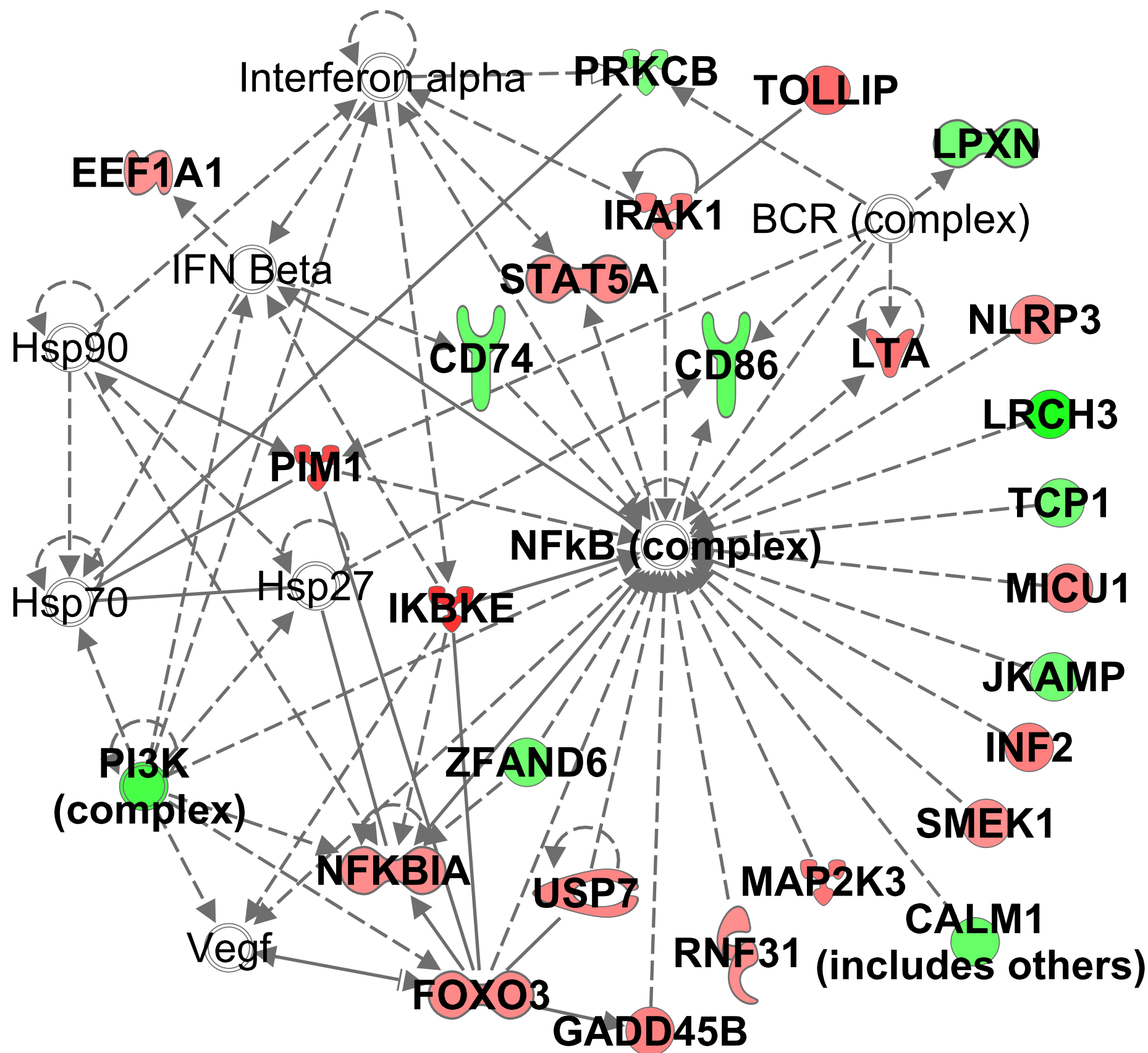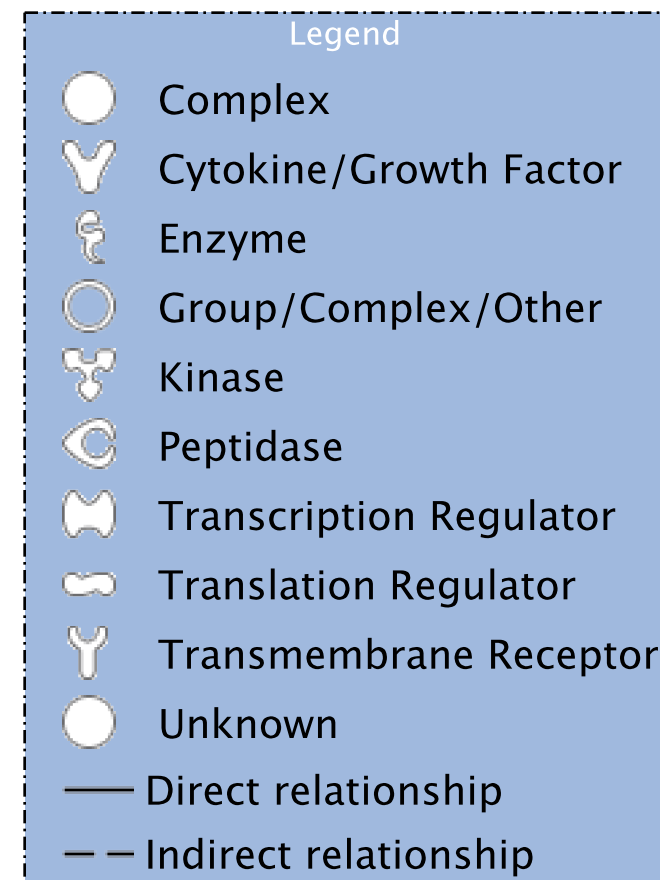

Supplement: Additional file 4: Figure S1 — Predictive accuracies based on the differences in deviance. The conventional markers-only model and the six prediction methods (50 splits into training and test datasets for each) were compared with the null model containing no covariates (the horizontal line at zero). A low value for the difference in deviance corresponds to a good predictive performance. The Lasso1, Ridge1 and BoostC1 models contain only the transcriptomic data, whereas the Lasso2, Ridge2 and BoostC2 models contain the transcriptomic data and the conventional predictors. [file 1755-8794-7-54-S4.pdf]

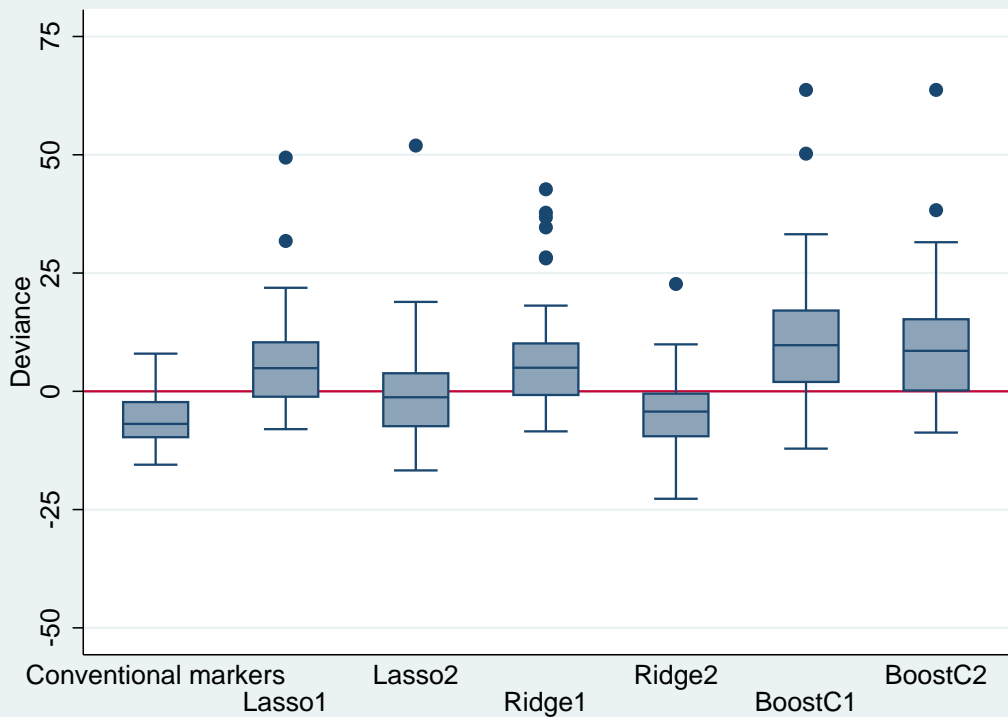

Supplement: Additional file 5: Figure S2 — Predictive accuracies based on the iRBS. The conventional marker-only model and the six prediction methods (50 splits into training and test data sets for each) were compared to the null model with no covariate (the horizontal line at zero). A large value for the difference in iRBS corresponds to a good prediction performance. The Lasso1, Ridge1 and BoostC1 models contain only the transcriptomic data, whereas the Lasso2, Ridge2 and BoostC2 models contain the transcriptomic data and the conventional predictors. [file 1755-8794-7-54-S5.pdf]

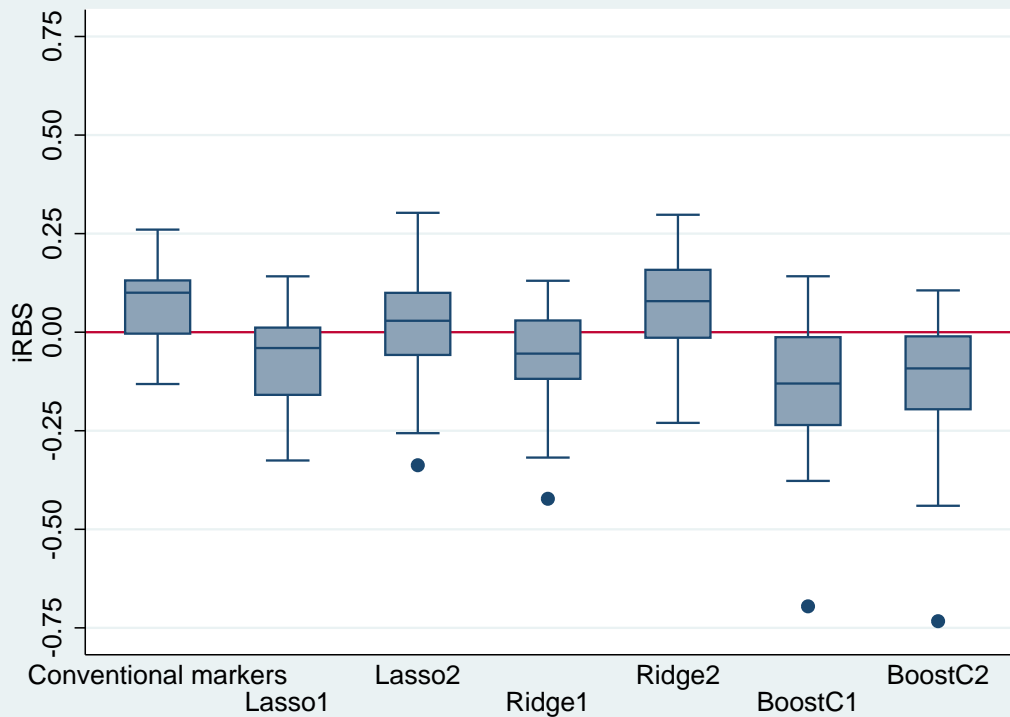

Supplement: Additional file 6: Figure S3 — The IPA-generated network based on the final signature transcripts. This network demonstrates that NF-κB is a prominent mediator of the molecular interconnections. The molecules incorporated into the final signature are shown in enlarged bold font, and the connective molecules are shown in regular font. Green color indicates that low expression level of the transcript predicts mortality, whereas red indicates that high expression level of the transcript predicts mortality. [file 1755-8794-7-54-S6.pdf]
