# Supplementary material for: Identification of a prognostic signature for old-age mortality by integrating genome-wide transcriptomic data with the conventional predictors: the Vitality 90+ Study
Source: BMC Med Genomics. 2014 Sep 11;7:54. doi: 10.1186/1755-8794-7-54 (PMC4167306; doi:10.1186/1755-8794-7-54)
Supplement: Additional file 7: Table S3 — Displaying the stepwise assessment of the variable combinations for the final Cox regression model. [file 1755-8794-7-54-S7.docx]

**Additional file 7: Table S3.** Stepwise assessment of the variable combinations for the final Cox regression model. Based on the AIC and Harrell’s *C*, the best model was model number 15.

| **Model #** | **Added variable(s)** | **Variables remaining in the model after stepwise selection** | **LL** | **AIC** | **Harrell’s *C*** |
| --- | --- | --- | --- | --- | --- |
| 1 | cf-DNA level, frailty index, BMI | cf-DNA level, frailty index, BMI | -220.8 | 449.5 | 0.711 |
| 2 | *KIR2DL1* | frailty index, BMI, *KIR2DL1* | -218.4 | 444.9 | 0.738 |
| 3 | *NME4* | frailty index, BMI, *KIR2DL1*, *NME4* | -212.8 | 435.6 | 0.771 |
| 4 | *MBP* | frailty index, BMI, *KIR2DL1*, *NME4*, *MBP* | -210.5 | 433.1 | 0.778 |
| 5 | *CHEK2* | cf-DNA level, frailty index, BMI, *KIR2DL1*, *NME4*, *MBP*, *CHEK2* | -206.5 | 429.0 | 0.794 |
| 6 | *AGAP1* | cf-DNA level, frailty index, BMI, *NME4*, *CHEK2*, *AGAP1* | -205.3 | 424.6 | 0.778 |
| 7 | *LRCH3* | frailty index, BMI, *KIR2DL1*, *NME4*, *CHEK2*, *AGAP1*, *LRCH3* | -196.0 | 408.1 | 0.804 |
| 8 | *RALGPS1* | frailty index, BMI, *KIR2DL1*, *NME4*, *CHEK2*, *AGAP1*, *LRCH3* | -196.0 | 408.1 | 0.804 |
| 9 | *IL1RN* | frailty index, BMI, *KIR2DL1*, *NME4*, *CHEK2*, *AGAP1*, *LRCH3* | -196.0 | 408.1 | 0.804 |
| 10 | *TMEM70* | frailty index, BMI, *KIR2DL1*, *NME4*, *CHEK2*, *AGAP1*, *LRCH3* | -196.0 | 408.1 | 0.804 |
| 11 | *GADD45B* | frailty index, BMI, *KIR2DL1*, *NME4*, *CHEK2*, *AGAP1*, *LRCH3* | -196.0 | 408.1 | 0.804 |
| 12 | *CDK6* | frailty index, BMI, *KIR2DL1*, *NME4*, *CHEK2*, *AGAP1*, *LRCH3* | -196.0 | 408.1 | 0.804 |
| 13 | *LTA* | frailty index, BMI, *KIR2DL1*, *NME4*, *CHEK2*, *LRCH3*, *TMEM70*, *LTA* | -191.6 | 401.1 | 0.835 |
| 14 | *SH2D1B* | frailty index, BMI, *KIR2DL1*, *NME4*, *CHEK2*, *LRCH3*, *TMEM70*, *LTA* | -191.6 | 401.1 | 0.835 |
| 15 | *VKORC1* | frailty index, BMI, *NME4*, *MBP*, *CHEK2*, *LRCH3*, *TMEM70*, *GADD45B*, *LTA*, *SH2D1B*, *VKORC1* | -183.6 | 391.1 | 0.857 |
| 16 | *IFRD2* | frailty index, BMI, *NME4*, *MBP*, *CHEK2*, *LRCH3*, *TMEM70*, *GADD45B*, *LTA*, *SH2D1B*, *VKORC1* | -183.6 | 391.1 | 0.857 |
| 17 | *LOC39157* | frailty index, BMI, *NME4*, *MBP*, *CHEK2*, *LRCH3*, *TMEM70*, *GADD45B*, *LTA*, *SH2D1B*, *VKORC1* | -183.6 | 391.1 | 0.857 |

Abbreviations: AIC, Akaike’s information criterion; BMI, body mass index; cf-DNA, cell-free DNA; LL, log likelihood
