# Supplementary material for: Identification of a prognostic signature for old-age mortality by integrating genome-wide transcriptomic data with the conventional predictors: the Vitality 90+ Study
Source: BMC Med Genomics. 2014 Sep 11;7:54. doi: 10.1186/1755-8794-7-54 (PMC4167306; doi:10.1186/1755-8794-7-54)
Supplement: Additional file 8: Table S4 — Displaying the results of the Ridge regression model performed with the conventional predictors and transcriptomic data (the combined model). [file 1755-8794-7-54-S8.docx]

**Additional file 8: Table S4.** Results of the Ridge regression model performed with the conventional predictors and transcriptomic data (the combined model). Only the 19 top-ranked variables exceeding the cut-off point 0.365 are displayed.

| **Variable** | **Gene name** | **Regression**  **coefficient** |
| --- | --- | --- |
| Frailty index |  |  |
| frail |  | 2.1090 |
| pre-frail |  | 1.5399 |
| cf-DNA level |  | 1.1461 |
| BMI |  | -0.1174 |
| *KIR2DL1* | *killer cell immunoglobulin-like receptor, two domains, long cytoplasmic tail, 1* | -0.0821 |
| *NME4* | *NME/NM23 nucleoside diphosphate kinase 4* | 0.0797 |
| *MBP* | *myelin basic protein* | -0.0732 |
| *CHEK2* | *checkpoint kinase 2* | -0.0577 |
| *AGAP1* | *ArfGAP with GTPase domain, ankyrin repeat and PH domain 1* | -0.0564 |
| *LRCH3* | *leucine-rich repeats and calponin homology domain containing 3* | -0.0489 |
| *RALGPS1* | *Ral GEF with PH domain and SH3 binding motif 1* | -0.0472 |
| *IL1RN* | *interleukin 1 receptor antagonist* | 0.0462 |
| *TMEM70* | *transmembrane protein 70* | -0.0430 |
| *GADD45B* | *growth arrest and DNA-damage-inducible beta* | 0.0411 |
| *CDK6* | *cyclin-dependent kinase 6* | 0.0408 |
| *LTA* | *lymphotoxin alpha (TNF superfamily, member 1)* | 0.0394 |
| *SH2D1B* | *SH2 domain containing 1B* | -0.0388 |
| *VKORC1* | *vitamin K epoxide reductase complex, subunit 1* | -0.0381 |
| *IFRD2* | *interferon-related developmental regulator 2* | 0.0367 |
| *LOC391578* | *MAF1 homolog (S. cerevisiae) pseudogene* | 0.0366 |

Abbreviations: BMI, body mass index, cf-DNA, cell-free DNA
